# Linking Pressure to Electrochemical Evolution in Solid-State Conversion Cathode Composites

**DOI:** 10.1021/acsami.5c20956

**Published:** 2025-12-31

**Authors:** Elif Pınar Alsaç, Arpan Kumar Sharma, Sun Geun Yoon, Bairav S. Vishnugopi, Congcheng Wang, Talia A. Thomas, Douglas Lars Nelson, Udochukwu D. Eze, Won Joon Jeong, John Harris, Partha P. Mukherjee, Matthew T. McDowell

**Affiliations:** † George W. Woodruff School of Mechanical Engineering, 1372 Georgia Institute of Technology, Atlanta, Georgia 30332, United States; ‡ School of Mechanical Engineering, 311308 Purdue University, West Lafayette, Indiana 47907, United States; § School of Materials Science and Engineering, Georgia Institute of Technology, Atlanta, Georgia 30332, United States

**Keywords:** solid-state batteries, conversion cathodes, stack pressure, sulfides, sulfur, pyrite

## Abstract

Conversion-type cathodes, such as sulfur, FeS_2_, and FeF_3_, offer high theoretical capacities in solid-state lithium batteries but are hindered by substantial volume changes during cycling, leading to interfacial contact loss, crack formation, and microstructural degradation. Here, we investigate the relationships between electrochemical, mechanical, and structural evolution in solid-state electrode composites with these three active materials. Using real-time stack-pressure monitoring, synchrotron X-ray absorption spectroscopy, and electrokinetic modeling, we elucidate how stress evolution is linked to reversible and irreversible redox reactions. Nonlinear stack pressure evolution in cells with sulfur, FeS_2_, and FeF_3_ electrode composites is found to arise from material-specific volume changes, the balance of volume change between the working and counter electrode, and the formation of distinct reaction intermediates. The three materials exhibit distinct stack pressure evolution, which is closely related to the different reaction processes in the materials, as demonstrated with X-ray absorption spectroscopy measurements. Through mesoscale modeling, we relate the experimental measurements to species evolution at the particle scale and track the dynamic coexistence of intermediate phases. Our findings highlight the importance of designing for volume changes of a given active material in solid-state battery systems.

## Graphical abstract

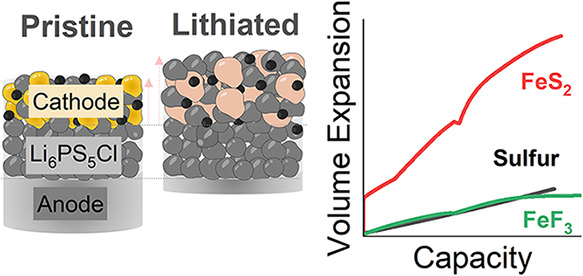

## Introduction

While lithium-ion batteries (LIBs) have long been a key energy storage technology, their dependence on flammable liquid electrolytes and their limited energy density pose challenges for meeting growing market demands.^1^ Solid-state batteries (SSBs) have emerged as a promising next-generation alternative, prompting the development of new materials and interfacial strategies for effective performance.^2,3^ Among cathode active materials (CAMs) for SSBs, intercalation-type oxides, such as LiNi_*x*_Mn_*y*_Co_1–*x*–*y*_O_2_ (NMC), have been extensively studied and are a leading candidate for practical applications due to their stability and reliable performance.^4,5^ However, their moderate theoretical capacities (∼160–200 mAh g^–1^), mechanical degradation, and high material costs pose barriers to large-scale deployment, motivating the investigation of alternative cathode active materials.^6–8^

Conversion type cathode materials, including sulfur,^9,10^ transition metal sulfides (e.g., FeS_2_ and CuS),^11–14^ and fluorides (e.g., FeF_2_ and FeF_3_),^15–18^ present a compelling alternative due to their high theoretical capacities enabled by multielectron redox reactions. Sulfur, FeS_2_, and FeF_3_ exhibit theoretical specific capacities of 1671, 894, and 712 mAh g^–1^, respectively. These materials show particular promise for SSBs because of the potential for enhanced stability at the electrolyte/electrode interface, as well as the elimination of the polysulfide shuttling effect when using solid electrolytes.^19–22^ Recent studies have also emphasized that the practical performance of pyrite-type cathodes is highly dependent on electrode design, including the conductive architecture, the active material particle size, and interfacial optimization, which are important considerations for high capacity cathode composite integration in SSBs.^23,24^

Despite these advantages, conversion cathodes present various challenges due to the insulating nature of material end-members (such as sulfur and Li_2_S), the need to maintain physical contact at the electrolyte interface,^25–27^ and the substantial volume changes of these materials, which can exceed 100% during lithiation/delithiation.^28–30^ Such drastic expansion and contraction can induce significant mechanical stress, leading to interfacial contact loss and/or fracture, which manifest distinctively in SSB systems compared to liquid-electrolyte batteries.^31,32^ Prior studies on conversion cathodes have highlighted how mechanical instabilities and microstructural evolution, in tandem with electrochemical reactions, play central roles in capacity fade.^33,34^ Additionally, the complex, multistep redox pathways in conversion materials can introduce uneven reaction kinetics and stress distributions, further exacerbating mechanical and electrochemical instabilities.^35–37^

Understanding electro-chemo-mechanical coupling in conversion cathode SSBs is essential for designing stable and long-lasting high-capacity SSBs. Previous investigations have shown that real-time stack pressure monitoring provides useful insights into mechanical evolution during cycling of a variety of types of SSB systems.^35,36,38–43^ The uniaxial stress applied to a cell stack (i.e., stack pressure) affects ion transport and interfacial contact.^44–46^ Moreover, changes of stack pressure in a constant-volume cell configuration also reflect the volumetric response of the active materials to lithiation/delithiation reactions, making it a key parameter for monitoring electro-chemo-mechanical coupling.^47,48^ These insights are particularly important for conversion-type cathodes whose redox reactions involve complex structural transformations and large volume changes. These materials often undergo multistep phase transitions that lead to the formation of intermediate and final products with different (partial) molar volumes and mechanical properties. FeS_2_ is particularly notable because it undergoes an irreversible conversion process during the first cycle, producing large and abrupt volume changes that can directly influence stack pressure evolution. As such, the evolution of internal stress is highly sensitive both to the kinetics of these reactions and the evolving electrode microstructure.

Here, we investigate the coupling between electrochemical transformations and stack pressure evolution in sulfur, FeS_2_, and FeF_3_ electrodes in SSBs. While these materials hold tremendous potential for next-generation SSBs, their electro-chemo-mechanical behavior presents critical challenges that necessitate advanced characterization and modeling to understand and address.^49,50^ We used real-time stack-pressure monitoring to track pressure changes and electrochemical signatures, and electron microscopy was used to correlate these factors to microstructure evolution in response to lithiation/delithiation of these materials. X-ray absorption fine structure (XAFS) spectroscopy revealed redox behavior at iron sites in FeS_2_ and FeF_3_, providing insight into the effects of transition metals on the chemo-mechanics of the conversion process. These results were further contextualized using microkinetic modeling, linking stack pressure evolution to the partial molar volume of intermediate phases and the dynamic coupling between electrochemical processes and mechanical stress. Our findings reveal how the distinct stack pressure evolution behaviors observed using sulfur, FeS_2_, and FeF_3_ cathodes are closely linked to their underlying redox pathways and microstructural transformations, underscoring the need for material-specific design strategies that mitigate mechanical degradation and enhance interfacial stability in high-capacity SSBs.

## Experimental Methods

### Cathode Composite Preparation

Conversion cathode composites were prepared by ball-milling cathode active material (CAM), Li_6_PS_5_Cl (LPSC, Ampcera, ∼1 μm particle size), and multiwalled carbon nanotubes (MWCNTs, Graphene Supermarket) with a CAM:LPSC:CNT weight ratio of 2:3:1 in a Fritsch Pulverisette 7 planetary ball mill. The CAMs used were sulfur (Thermo Scientific, 325 mesh, 99.5%), FeS_2_ (Sigma-Aldrich, 325 mesh, 99.8% trace metals basis), or FeF_3_ (Thermo Scientific, anhydrous, 97% min., metal basis). Approximately 1.2 g of the mixture of CAM, LPSC, and MWCNTs was added to a ZrO_2_ jar with four 10-mm diameter ZrO_2_ balls and 12 g of 1-mm diameter ZrO_2_ balls using a 1:20 weight ratio between the composite and ball weight. The mixture was prepared and sealed in the jar within an Ar-filled glovebox before the milling process. Milling was performed at 500 rpm for 24 cycles, with milling for 10 min and resting for 5 min per cycle. After milling, the jar was opened in an Ar-filled glovebox to collect the composite powder.

### Counter Electrode Fabrication

Various counter electrodes were used in different cells. Counter electrodes consisting of 20-μm lithium on a 10-μm copper current collector backing (MSE Supplies) were used as-received. A different counter electrode consisted of a mixture of single-crystalline LiNi_0.6_Mn_0.2_Co_0.2_O_2_ (NMC622) coated with LiNb_0.5_Ta_0.5_O_3_ (LNTO) as the active material (MSE Supplies), LPSC (Ampcera, ∼1 μm particle size), and vapor grown carbon fiber (VGCF, Sigma-Aldrich). LNTO was fabricated and coated on the NMC622 cathode active material before composite preparation to lessen side reactions between the solid electrolyte and the cathode active material. Stoichiometric ratios of lithium acetate (LiCO_2_CH_3_, Sigma-Aldrich, 99.95%), tantalum butoxide (Ta­(OCH_2_CH_2_CH_2_CH_3_)_5_, Sigma-Aldrich, 99.99%) and niobium ethoxide (Nb­(OCH_2_CH_3_)_5_, Sigma-Aldrich, 99.95%) were dissolved in dry ethanol (Sigma-Aldrich, 99.5%) and stirred for 12 h. NMC622 powder was added to this solution and mixed using ultrasonication (Branson 1510 Ultrasonic Cleaner at 40 kHz for 2 h at 25 °C). Then the solvent was evaporated in a vacuum oven for 8 h. 1 mL of solution was added to 2 g of NMC622 powder, followed by heating to 450 °C for 1 min in air. The final composition of the composite was 70 wt % of LNTO-coated NMC622, 27.5 wt % LPSC, and 2.5 wt % VGCF. The mixture was ball-milled (Fritsch Pulverisette 7) at three cycles of 150 rpm for 15 min of milling and 5 min of resting in a ZrO_2_ milling jar with eight 1 mm-diameter ZrO_2_ milling balls. The jar was sealed and opened after milling in an Ar-filled glovebox.

### Solid-State Cell Assembly

90 mg of LPSC powder was loaded in a 10 mm diameter die made from polyether ether ketone (PEEK) and pelletized under uniaxial compression at 150 MPa for 1 min. The sulfur/FeS_2_/FeF_3_ composite was loaded and repressed at 375 MPa for 5 min and lithium foil was then attached to the opposite side, and the cell was repressed at 60 MPa for 10 min. For the cells prepared with NMC counter electrodes, a two-step pressing protocol was followed. Here, the sulfur/FeS_2_/FeF_3_ composite and the NMC composite were loaded on top and below the LPSC pellet and pressed at 375 MPa for 10 min. We used 2.0–3.4 mg of active material loading depending on the theoretical specific capacity of the cathode material. To ensure mechanically stable counter electrodes, different areal capacities were selected for Li metal and NMC622. A 20-μm Li foil was used (corresponding to ∼4 mAh cm^–2^) because this thickness can sustain stack pressure without excessive plastic deformation, whereas thicker Li foils exhibit exacerbated deformation.^51^ In contrast, the NMC622 counter electrode featured 6.0 mAh cm^–2^ to operate within a volume-stable regime, thereby minimizing volume changes at this counter electrode.^52^ These design choices prevent Li depletion at the solid-electrolyte interface and mitigate interfacial mechanical degradation, enabling representative electrochemical behavior for both cell configurations. A stack pressure of approximately 21 MPa was applied followed by resting for 6 h. All the cells were cycled in an Ar-filled glovebox.

### Stress Measurements

Stress measurements were performed in accord with the previously reported procedure.^38^ A custom-built cell assembly was used which included a force sensor integrated underneath the cell stack physically separated from the Ti rod using a PEEK plate. The force sensor (2 kN, KMM50, Inelta Sensorsysteme) was paired with a signal conditioner (IMA2-DMS, Inelta Sensorsysteme). The simultaneous electrochemical response was recorded using a Landt CT2001 battery tester. The pressure change during cell cycling was recorded with Python. The time derivative of the stress curves (d*P*/d*t*) and d*Q*/d*V* curves were calculated with OriginLab using Savitsky-Golay smoothing, followed by linear interpolation and differentiation. The cells with Li counter electrodes were cycled between 1.4 and 3.0 V, and the cells with NMC counter electrodes were cycled between −2.5 and 0 V. All of the cells were cycled using 0.1 mA cm^–2^ current density. The areal capacity loading was 4 mAh cm^–2^ for the Li cells and 6 mAh cm^–2^ for the NMC cell. The cells were rested at open circuit for 6 h prior to electrochemical testing.

### X-ray Absorption Spectroscopy (XAS)

The *ex situ* XAS experiments on FeS_2_ and FeF_3_ were performed at the 6-BM Beamline for Materials Measurements (BMM) at the National Synchrotron Light Source II (Brookhaven National Laboratory, NY) using a Si(111) monochromator. Data was collected in fluorescence mode for the Fe K-edge using a four-element Si drift detector. The Athena software package was used for data reduction of the XAS spectra using standard processes.^53^ The Artemis software package was used to generate structural models by simulating k^3^-weighted extended X-ray absorption fine structure (EXAFS) results between k values of 3 and 12.5 Å^–1^. The location of the Fe K-edges was determined by the half-height method. MorletE software was used for wavelet transform analysis. The cells were run to different states of charge or discharge and then removed from the cells. The disassembled pellets were prepared and sealed in a polymer-covered aluminum pouch cell material to prevent air ingress. The sample surface was covered with Kapton tape as a window to increase the flux of the beam. Samples were prepared in the pristine state, after the first discharge, and after the first charge.

### Scanning Electron Microscopy (SEM)

SEM images were collected with a Hitachi SU8230 SEM using an accelerating voltage of 10 kV and a working distance of 8 mm. The cells were sliced with a scalpel to investigate the cross sections of the electrodes. During loading into the SEM, the samples were exposed to the atmosphere for less than 10 s, and evacuation of the loading chamber took around 30 s.

### Mechanistic Modeling

The complex, nonlinear electrochemical behavior of conversion cathode cells arises from the coupled effects of interfacial reaction kinetics, ionic transport through the solid electrolyte, and spatially nonuniform species evolution within the composite cathode. These processes span multiple length scales, ranging from particle-scale to the porous electrode microstructure scale, and they are described by the following conservation and reaction equations.^54–56^

The charge conservation equation in the CAM and solid electrolyte (SE) phase is described in eq 1:

1 $$\nabla i_{\mathrm{SE}}+\nabla i_{\mathrm{CAM}}=0$$

Here, *i* is the current density in the phase. The distribution of electric potential distribution in the CAM and SE phases is given in eqs 2 and 3:

2 $$\nabla \cdot \left(\sigma_{s}^{\mathrm{eff}}\nabla \phi_{s}\right)-a_{s}i_{\mathrm{CAM}}=0$$

3 $$\nabla \cdot \left(\kappa_{s}^{\mathrm{eff}}\nabla \phi_{e}\right)+a_{s}i_{\mathrm{SE}}=0$$

We assume the following reaction:

$$A\to B+ne^{-}+nC^{-}$$

Here, *A*, *B* and *C* are the species involved in the reaction. The charge transfer reaction rate can be estimated using the Butler–Volmer equation (eq 4, with overpotential defined in eq 5):

4 $$i=\mathrm{nFk}\left\{\varepsilon_{A}^{*}\;\exp\left(\beta \frac{F}{\mathrm{RT}}\eta \right)-\varepsilon_{B}^{*}\;\exp\left(-\left(1-\beta \right)\frac{F}{\mathrm{RT}}\eta \right)\right\}$$

5 $$\eta =\phi_{S}-\phi_{E}-E^{0}$$

Here, *k* is the reaction rate coefficient, ϵ* is the normalized volume fraction (0 < ϵ* < 1) of solid species in the electrode, *E* ^**0**^ is the reaction’s standard potential vs Li/Li^+^, η is the overpotential, and β is the symmetry factor for the reactions. The normalized volume fraction is calculated by dividing the species volume fraction with the initial volume fraction. As the reaction proceeds, the rate of consumption and generation of species can be given as shown in eqs 6 and 7:

6 $$\frac{\partial \varepsilon_{A}}{\partial t}=-\frac{a_{s}i}{\mathrm{nF}}V_{A}$$

7 $$\frac{\partial \varepsilon_{B}}{\partial t}=\frac{a_{s}i}{\mathrm{nF}}V_{B}$$

Here, *V*_*i*_ is the molar volume of the species, *a*_s_ is the specific active area, and *n* is the number of electrons transferred during the reaction.

The volume expansion (%) of the cathode is calculated as eq 8:

8 $$\Delta V=\frac{{(\sum_{1}^{n}V_{i}\epsilon_{i})}_{\mathrm{final}}-{\left(\sum_{1}^{n}V_{i}\epsilon_{i}\right)}_{\mathrm{initial}}}{{\left(\sum_{1}^{n}V_{i}\epsilon_{i}\right)}_{\mathrm{initial}}}\times 100$$

As a first approximation, we assume that the volume change does not alter the electrode microstructural properties.

## Results and Discussion

### Electrochemical Behavior

The electrochemical behavior of sulfur, FeS_2_, and FeF_3_ electrode composites was investigated at room temperature in anvil-type solid-state cells. These cells were cycled at a constant current density of 0.1 mA cm^–2^ between 1.4 and 3.0 V vs. Li/Li^+^, they featured a stack pressure of 20 MPa, they used an argyrodite-type Li_6_PS_5_Cl (LPSC) solid electrolyte, and they contained a 20-μm thick lithium foil counter electrode. The cathode active material (CAM) loading corresponded to an areal capacity of 4.0 mAh cm^–2^. The lithiation-delithiation profiles (Figure 1a–c) show distinct behavior for each active material. The sulfur cathode (Figure 1a) achieved an areal capacity of 1.7 mAh cm^–2^ upon initial lithiation, equivalent to a specific capacity of 675 mAh g_sulfur_^–1^. This is only 42.5% of the theoretical capacity; this relatively low capacity is expected for sulfur cathodes operated at room temperature without structural or morphological modifications and moderate stack pressures. In other studies, stack pressures for these types of cells have varied between 50 to a few hundred MPa.^12,30,48,50,57,58^ In comparison, FeS_2_ and FeF_3_ demonstrated higher initial areal lithiation (discharge) capacities of 2.5 and 2.0 mAh cm^–2^, respectively, with corresponding specific capacities of 628 mAh g_FeS_2__^–1^ and 535 mAh g_FeF_3__^–1^ (Figure 1b,c). Both FeS_2_ and FeF_3_ exhibit higher fractional capacity of theoretical compared to the sulfur composite (70.5% and 75.2%, respectively). Similar cells cycled with LiNi_0.6_Mn_0.2_Co_0.2_O_2_ (NMC622) counter electrodes exhibited slightly higher initial specific capacities of 855 mAh g_sulfur_^–1^, 697 mAh g_FeS_2__^–1^, and 695 mAh g_FeF_3__^–1^, which could be attributed to better interfacial contact between the Li_6_PS_5_Cl separator and the counter electrode during the application of formation pressure and densification of composites^59^ (Figure S1).

**Figure 1:**
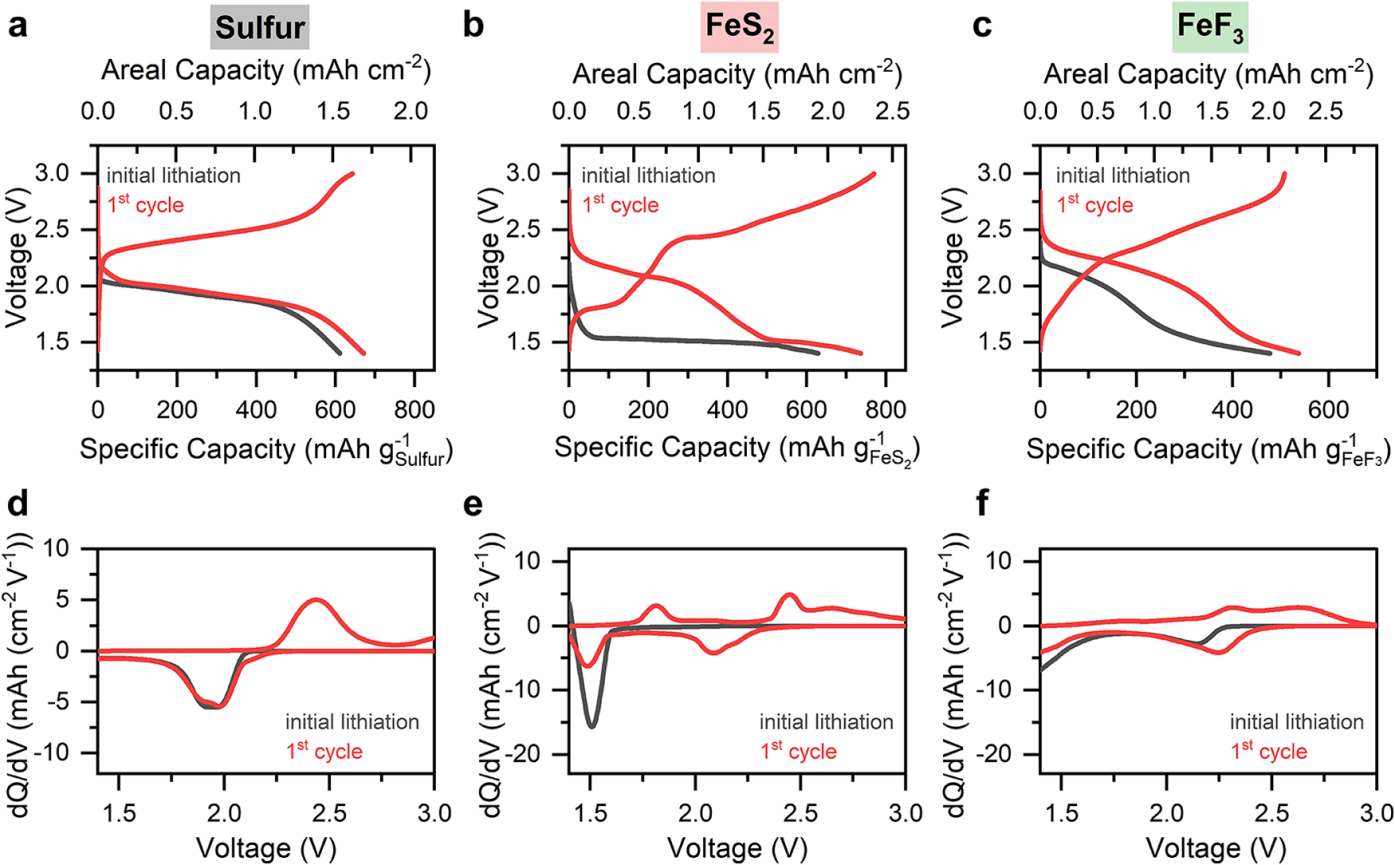
Electrochemical behavior of sulfur, FeS_2_, and FeF_3_ composite electrodes in solid-state cells with Li_6_PS_5_Cl solid electrolyte and Li counter electrodes. The experiments were carried out at room temperature using 0.1 mA cm^–2^ current density and 20 MPa stack pressure. (a–c) Charge–discharge profiles of cells with (a) sulfur, (b) FeS_2_, and (c) FeF_3_ electrode composites. (d–f) Corresponding differential capacity (dQ/dV) curves for each cell in panels a–c.

The dQ/dV curves derived from the sulfur composite data (Figure 1d) provide detailed insights into the redox reactions during the initial lithiation and subsequent first full cycle. The initial lithiation dQ/dV curve from the sulfur composite shows a similar shape compared to the following lithiation. A broad discharge peak with two small features at 1.95 and 2.05 V is observed in both discharges, while the peak at 2.05 V is more pronounced in the initial lithiation likely due to activation of sulfur and surface reconstruction.^25^ The peak located at 2.05 V is attributed to the reduction of sulfur to Li_2_S_2_, while the second corresponds to the formation of the final lithiation product (Li_2_S), although these processes are likely overlapping.

The cells containing FeS_2_ and FeF_3_ electrodes show greater differences in their voltage profiles between the initial lithiation and the following cycle, which reflects chemical and structural changes during the initial conversion reaction upon lithiation (Figure 1b,c and e,f). For FeS_2_, the peak in the dQ/dV data at ca. 1.55 V represents the conversion of FeS_2_ to Li_2_S and metallic iron species. During the first delithiation, two peaks appear at 1.8 and 2.4 V. The first peak is attributed to the reaction between metallic iron and Li_2_S, forming FeS, while the second involves conversion of Li_2_S to sulfur (Figure 1e). The FeF_3_ cell features one peak at 2.25 V during the initial lithiation, attributed to the intercalation of lithium into FeF_3_, followed by the formation of LiFeF_3_ (Figure 1f). A conversion reaction occurs at 1.8 V, which leads to the formation of LiF and metallic iron species (Table 1).^60^

**Table 1:** Reaction Processes and Corresponding Equilibrium Potentials (in V vs. Li/Li^+^) for Sulfur, FeS_2_ and FeF_3_ Electrodes Based on Previous Studies^33,57,60,65,66^

**reaction**	***E*** ^**0**^	**reaction**	***E*** ^**0**^
**sulfur**			
**lithiation**		**delithiation**	
R1 $S_{8}+8Li^{+}+8e^{-}\to 4Li_{2}S_{2}$	2.1	R3 $8Li_{2}S\to 16Li^{+}+16e^{-}+S_{8}$	2.7
R2 $Li_{2}S_{2}+2Li^{+}+2e^{-}\to 2Li_{2}S$	2.05		
**FeS**_**2**_			
**initial lithiation**		**delithiation**	
R4 $\mathrm{Fe}S_{2}+4Li^{+}+4e^{-}\to 2{\mathrm{Li}}_{2}S+\mathrm{Fe}$	1.85	R7 $\mathrm{Fe}+Li_{2}S\to \mathrm{FeS}+2{\mathrm{Li}}^{+}+2e^{-}$	1.8
		R8 $8Li_{2}S\to S_{8}+16{\mathrm{Li}}^{+}+16e^{-}$	2.4
**subsequent lithiation steps**			
$\mathrm{Fe}S+2xLi^{+}+2xe^{-}\to {\mathrm{Li}}_{2x}\mathrm{Fe}S$ R5	2.2		
${\mathrm{Li}}_{2x}\mathrm{Fe}S+(2-2x){\mathrm{Li}}^{+}+(2-2x)e^{-}\to \mathrm{Fe}+Li_{2}S$ R6	1.4		
**FeF**_**3**_			
**lithiation**		**delithiation**	
R9 $\mathrm{Fe}F_{3}+Li^{+}+e^{-}\to \mathrm{LiFe}F_{3}$	2.2	R11 $\mathrm{Fe}+3\mathrm{LiF}\to \mathrm{LiFe}F_{3}+2Li^{+}+2e^{-}$	2.0
R10 $\mathrm{LiFe}F_{3}+2Li^{+}+2e^{-}\to \mathrm{Fe}+3\mathrm{LiF}$	1.8	R12 $\mathrm{LiFe}F_{3}\to \mathrm{Fe}F_{3}+Li^{+}+e^{-}$	2.6

### Stack Pressure Evolution

The selection of these three conversion materials was motivated by their distinct conversion reactions during initial lithiation, as demonstrated in Figure 1. These different reactions involve varying volume changes. FeF_3_ undergoes an initial intercalation reaction followed by a two-electron conversion reaction, with a volume increase of 25% during the full lithiation process.^60^ In contrast, the conversion of S_8_ to 8Li_2_S involves a 16-electron transfer reaction and generally occurs as a two-step reaction, which includes the formation of a metastable Li_2_S_2_ phase followed by conversion to the Li_2_S phase, resulting in a volume increase of 80%.^28^ FeS_2_ undergoes a two-step, four-electron transfer reaction during the initial lithiation, leading to a substantial volume increase of 159%.^29^

To examine how electrochemical behavior is linked to stack pressure evolution and volume changes in these conversion cathodes, a custom solid-state cell assembly with an integrated force sensor was used (see Experimental section). This cell approximates a constant volume environment, with the polyether ether ketone (PEEK) casing and metal rods held in place without springs and the force sensor measuring changes in force due to volume changes of the active materials. Cells with sulfur, FeS_2_, or FeF_3_ composite electrodes were used with either lithium or NMC622 counter electrodes (Figure 2).^39^

**Figure 2:**
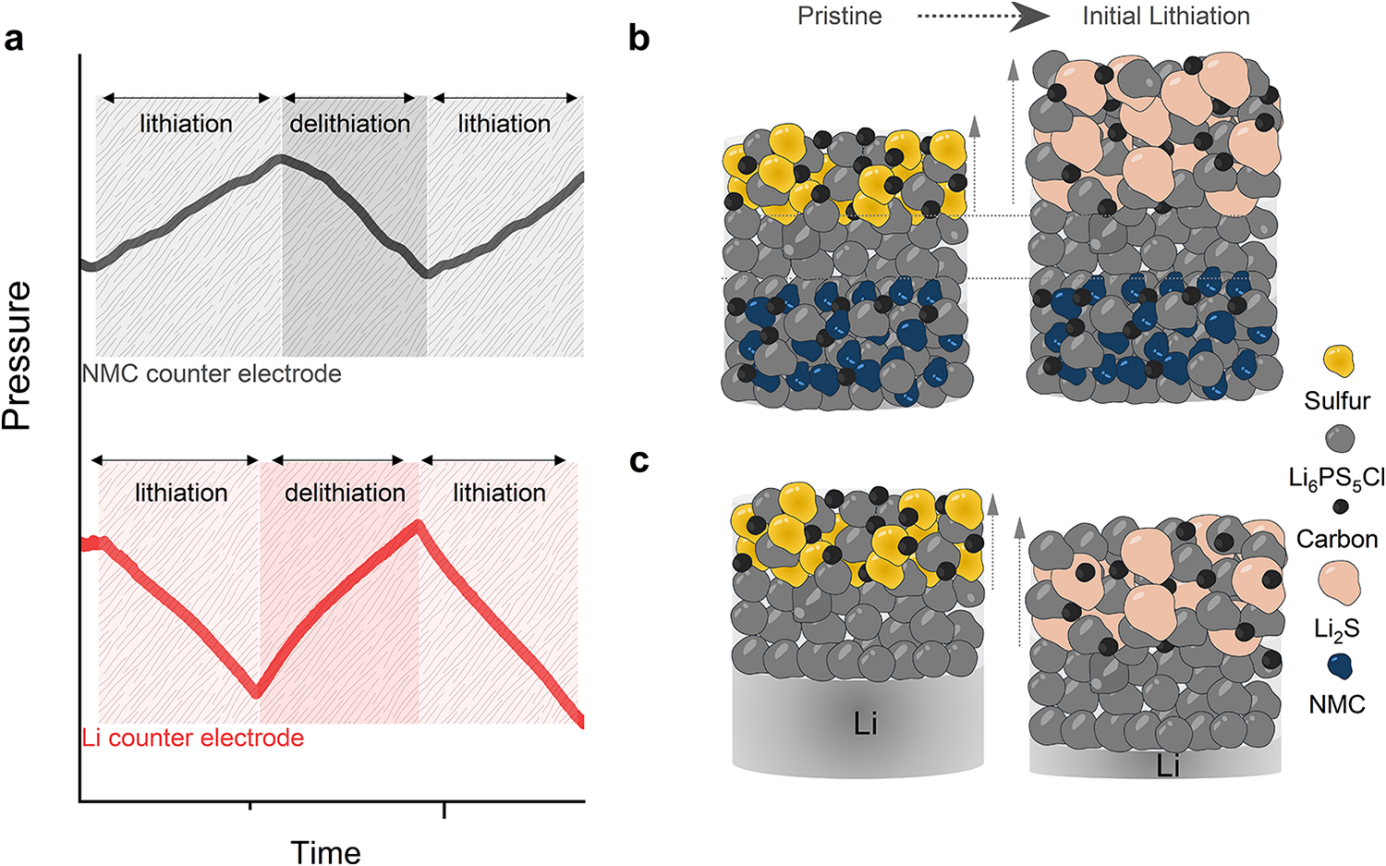
Pressure evolution in cells with conversion cathodes with different counter electrodes. (a) Typical experimental data in a constant-volume cell showing pressure change during lithiation, delithiation, and second lithiation when using composite conversion cathodes paired with different counter electrodes: NMC622 counter electrode (black) and lithium metal counter electrode (red). (b–c) Schematics of the volume changes of the cell stacks with (b) NMC622 and (c) lithium counter electrodes. The balance of the partial molar volume of lithium species in each electrode determines the net volume change, with a net increase in stack volume (increasing stack pressure) during lithiation when using an NMC622 counter electrode and a net decrease in stack volume (decreasing stack pressure) during lithiation when using a lithium counter electrode.

Figure 2 depicts the distinct volume change and stack pressure evolution behaviors of these conversion electrode materials when paired with different counter electrodes. When lithium metal is used as the counter electrode, its large volume changes during cycling due to the high molar volume of Li dominate the overall pressure evolution,^52,61^ effectively masking pressure contributions from the conversion cathode composite (Figure 2a). As a result, the overall stack pressure decreases during lithiation due to the net volume decrease of the cell stack (Figure 2c), and the greater volume change of Li compared to the conversion cathode makes it difficult to isolate the individual contributions of the cathode composite from the overall stack pressure change. In contrast, using an intercalation-type material as the counter electrode allows for the conversion cathode to play a greater role in the net stack volume evolution. Intercalation materials feature smaller partial molar volumes of Li^+^ and thus undergo smaller volume changes (typically in the range of 2–5%) compared to lithium metal.^62^ This reduced volume change minimizes the impact of the counter electrode on the overall pressure evolution, allowing the chemo-mechanical behavior of the conversion cathode composites to be more clearly observed and studied (Figure 2b). The pressure evolution in these cells is thus predominantly governed by the conversion cathode, as the counter electrode contribution is minimized. As shown in Figure 2a, this results in a net pressure increase during lithiation due to the net volume change of the stack being controlled by the conversion cathode, which expands upon lithiation. The cell configuration with an NMC622 counter electrode allows for more detailed investigation of pressure-reaction linkages for the conversion cathode, and the overall trends in Figure 2 highlight the need to consider the (partial) molar volume balance of Li species within the electrodes in determining volume changes and pressure evolution.

The experimental results in Figure 3a–c show voltage traces (top) and measured stack pressure changes (bottom) over three cycles for cells containing sulfur (Figure 3a), FeS_2_ (Figure 3b), and FeF_3_ (Figure 3c), along with 20-μm thick Li metal counter electrodes. The initial stack pressure was set to approximately 21 MPa. In all cases, the stack pressure evolution during cycling exhibits a clear cyclic pattern, in which it decreases during lithiation of the active material and increases during delithiation, which reflects the net volume contraction of the stack upon lithiation of the cathode and net volume expansion of the stack during delithiation (as shown in Figure 2). This behavior is consistent across the three materials, although the magnitudes and shapes of the pressure responses vary due to differences in reaction mechanisms and volume change characteristics (to be discussed subsequently). The cell with FeF_3_ exhibited the greatest pressure changes, with a 1.1 MPa decrease during initial lithiation, while the cell with FeS_2_ showed the lowest (0.6 MPa).

**Figure 3:**
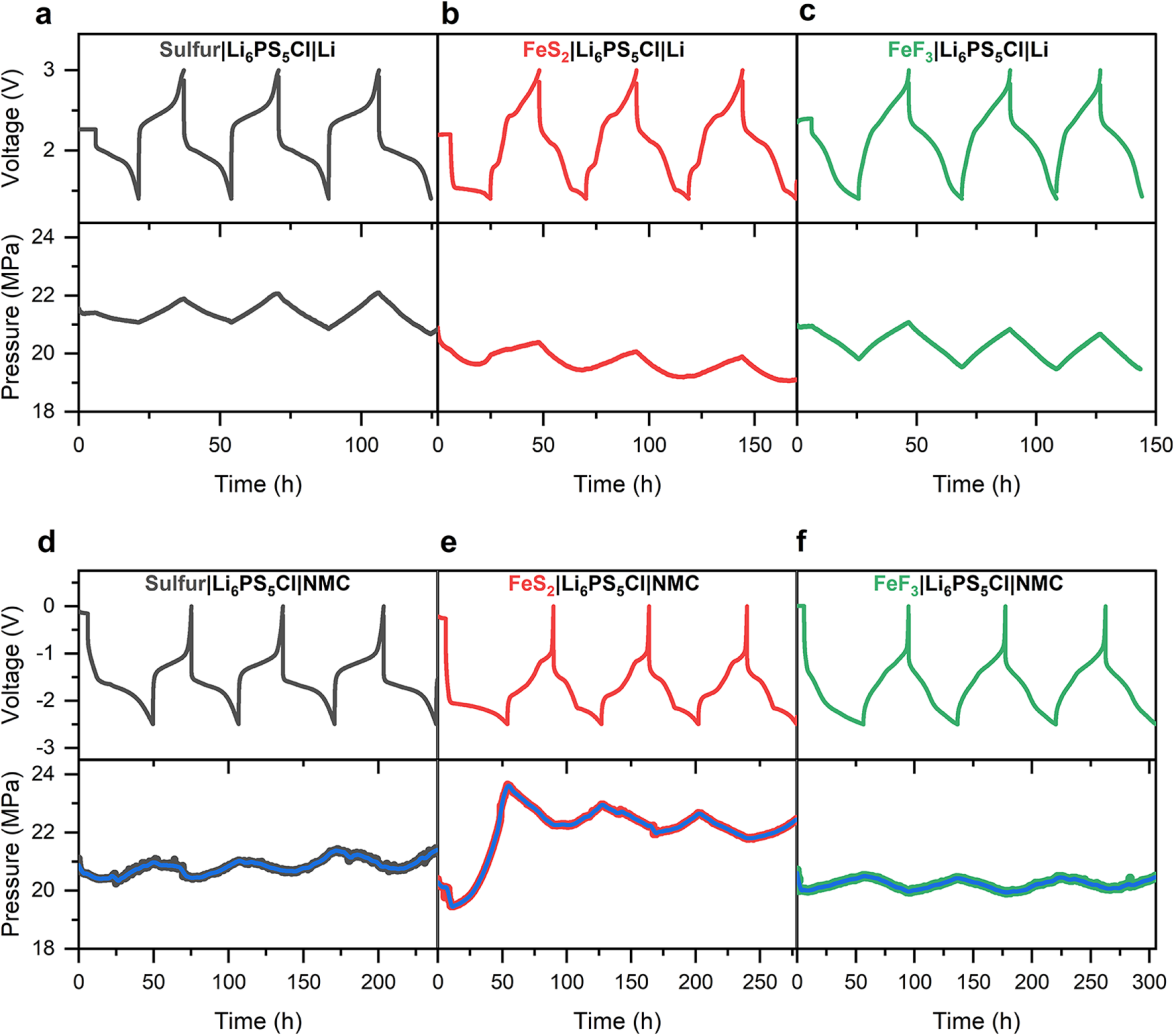
Galvanostatic voltage profiles (top) coupled with in situ stack pressure measurements (bottom) of composite conversion material working electrodes with different counter electrodes in solid-state cells with Li_6_PS_5_Cl solid electrolyte. (a–c) Sulfur (a), FeS_2_ (b), and FeF_3_ (c) composite working electrodes in cells with 20 μm-thick Li negative electrodes. (d–f) Sulfur (d), FeS_2_ (e), and FeF_3_ (f) composite working electrodes in cells with NMC622 composite counter electrodes. The blue lines are smoothened using a Savitzky-Golay filter. Each cell was tested with a current density of 0.1 mA cm^–2^ at 25 °C.

The cells cycled with NMC622 counter electrodes (Figure 3d–f) show pressure evolution trends that are opposite to those observed with a lithium metal counter electrode; specifically, there is an increase in stack pressure during lithiation and a decrease during delithiation. The cell with a sulfur electrode shows a 0.8 MPa stack pressure increase during the first lithiation (Figure 3d). There were slightly higher pressure changes for sulfur in the following cycles due to higher capacity in later cycles related to activation of additional sulfur.^63^ FeS_2_ exhibits a substantial stack pressure increase of about 4 MPa during the initial lithiation, followed by smaller (∼1 MPa) cyclic pressure changes at higher stack pressure during cycling (Figure 3e). The large pressure change on the first lithiation indicates much larger volume change and a distinct conversion reaction pathway on the first discharge compared to further cycling. This finding is related to the different electrochemical signatures during the first vs. later lithiation processes for FeS_2_ (Figures 1b and 3e), suggesting different reaction pathways. Interestingly, the FeS_2_ cell with a lithium counter electrode (Figure 3b) shows only a minimal pressure change on the first lithiation, indicating that the volume expansion of the FeS_2_ electrode is mostly negated by the contraction of the lithium counter electrode. The cell with a FeF_3_ working electrode and NMC counter electrode shows a cyclic stack pressure change of ∼0.5 MPa (Figure 3f). Finally, it should be noted that the magnitude of pressure change is dependent on the initial stack pressure values as well as the details of the cell assembly and configuration, and differences are therefore expected when comparing these magnitudes to other studies. For instance, Lee et al. observed approximately 4 MPa change in pressure with 70 MPa applied stack pressure for a sulfur cathode.^50^ However, direct comparison of the different materials within the current study using identical cell configurations provides useful insight.

### Pressure Hysteresis and Slope

Figure 4a presents the stack pressure evolution as a function of normalized capacity for cells with sulfur, FeS_2_, and FeF_3_ cathodes in the first and third cycles using Li metal counter electrodes. A 20-μm lithium film on Cu was used to avoid excess flow of thicker Li foils under stack pressure, and it avoids phase inhomogeneities often observed with lithium–indium alloy counter electrodes.^51,64^ During the first cycle, the sulfur and FeS_2_ cathodes show smaller variations in stack pressure than FeF_3_. This is likely because FeF_3_ undergoes the smallest intrinsic volume change among the three cathodes during its conversion reaction, making the overall stack pressure more strongly influenced by the large volume changes of the lithium counter electrode, which dominate pressure evolution of the cell stack. By the third cycle, both sulfur and FeS_2_ show higher stack pressure changes compared to the first cycle, which can be attributed to the gradual activation and improved electrochemical utilization of these materials. In all three systems, the slope of the pressure response changes as capacity increases, particularly toward the end of lithiation.

**Figure 4:**
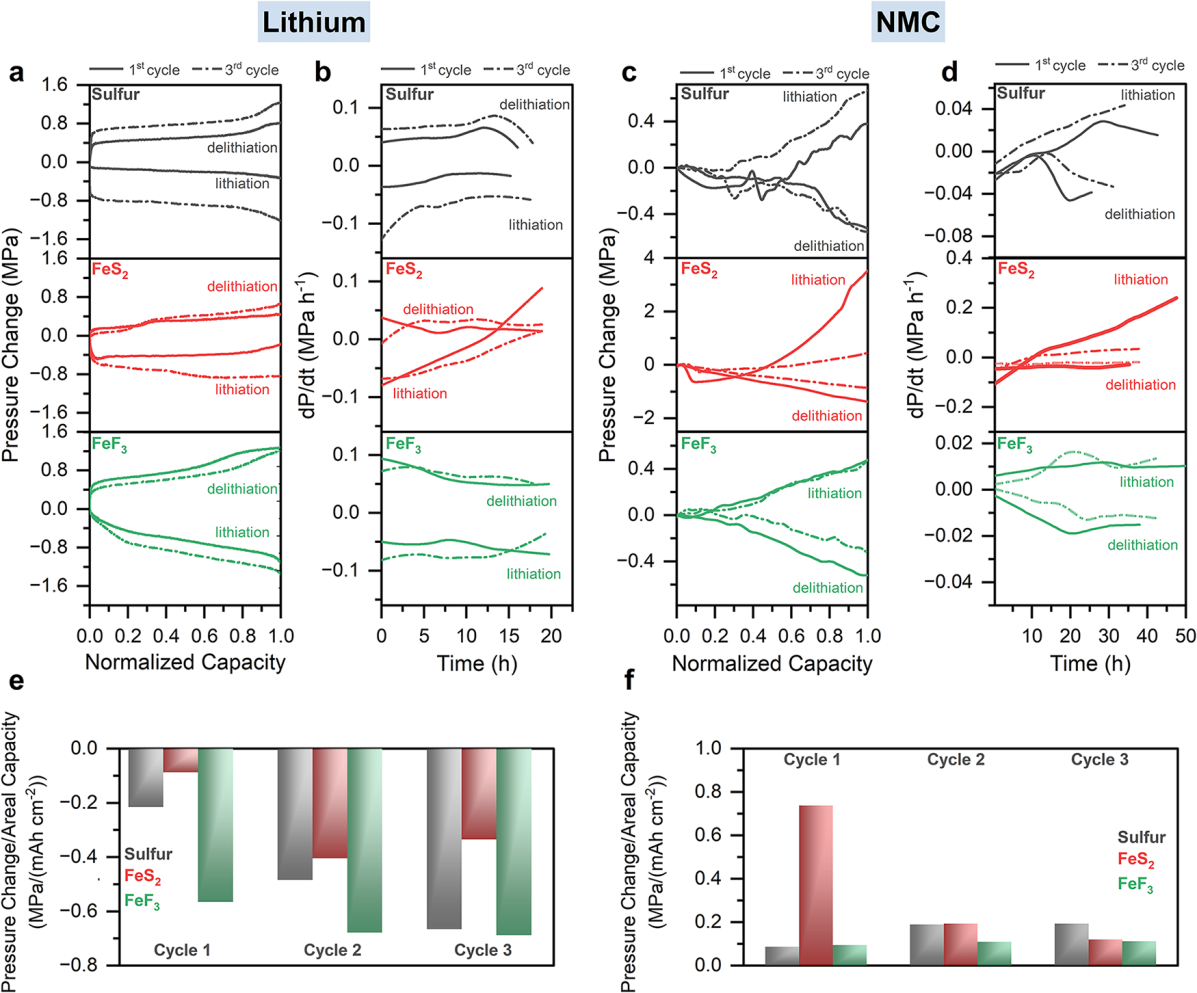
Pressure hysteresis and d*P*/d*t* analysis during cell cycling. (a) Stack pressure changes of cells featuring the composite conversion electrodes with lithium counter electrodes with respect to capacity normalized by the maximum capacity and (b) corresponding differential analysis (d*P*/d*t* curves) of the stack pressure evolution of cells with sulfur **(top)**, FeS_2_ **(middle)**, and FeF_3_ **(bottom)**. (c) Stack pressure changes of cells featuring the composite conversion electrodes with NMC counter electrodes with respect to capacity normalized by the maximum capacity and (d) corresponding differential analysis (d*P*/d*t* curves) of the stack pressure evolution of cells with sulfur **(top)**, FeS_2_ **(middle)**, and FeF_3_ **(bottom)**. Stack pressure change normalized by the areal capacity of the cells over the first three cycles for sulfur (gray), FeS_2_ (red), and FeF_3_ (green) in cells with lithium counter electrodes (e) and NMC counter electrodes (f).

Figure 4b shows the corresponding time derivatives of the stack pressure (d*P*/d*t*) for each of the conversion cathode cells in Figure 4a. The d*P*/d*t* curves provide insight into how lithiation and delithiation dynamics evolve over time. They can also reveal changes of the effective partial molar volume during half-reactions since the time axis is a proxy for charge passed in these galvanostatic experiments. Previous studies on silicon^38^ and graphite^42^ anodes have correlated stack pressure changes to structural evolution of the electrodes by using such d*P*/d*t* analysis during electrochemical cycling.

The d*P*/d*t* curves of cells containing sulfur, FeS_2_ and FeF_3_ electrodes with Li counter electrodes (Figure 4b) show that the slopes of the pressure changes are not necessarily constant throughout the lithiation/delithiation processes. Similar nonconstant behavior is observed for the materials when using NMC counter electrodes (Figure 4c,d). Varying d*P*/d*t* values indicate that the volume change varies with state of charge (SOC). This can be due to SOC-dependent partial molar volume of Li ( $\overset{®}{V_{m}}$ ) within the conversion material, or to the effects of porosity within the composite electrode that can accommodate some of the expanding volume and therefore reduce the exerted force. Of note, the cell with the FeS_2_ working electrode and NMC counter electrode (Figure 4d) exhibited a substantial (∼4 MPa) pressure increase during the first lithiation, with the d*P*/d*t* curve also continuously increasing during the lithiation. There is a significant volume expansion of the FeS_2_ cathode during this first lithiation, which is then lessened on subsequent cycling. Since this is a continuous conversion reaction to form Li_2_S and metallic iron particles with theoretically constant $\overset{®}{V_{m}}$ , the increasing slope with lithiation suggests that the expanding volume progressively fills pores within the composite, gradually exerting higher forces as the pores are filled. Furthermore, the initially negative d*P*/d*t* of the sulfur composite during lithiation (Figure 4c,d) also suggests pore filling during initial expansion, which may be due to highly distributed sulfur within the composite electrode. In contrast, FeF_3_ features positive d*P*/d*t* values during lithiation, indicating that the lithiation-induced expansion directly translates into measured stack pressure. These findings emphasize the complex interplay between microstructural changes and partial molar volume ( $\overset{®}{V_{m}}$ ) variations in determining the internal pressure evolution within SSBs.

Analysis of the stack pressure normalized by the areal capacity of the cells, both with lithium counter electrodes (Figure 4e) and NMC counter electrodes (Figure 4f), allows for comparisons of overall stack pressure evolution while removing differences in specific capacity of the cells. With lithium counter electrodes (Figure 4e), the cells with FeF_3_ electrodes show the largest normalized stack pressure changes over each cycle, which is likely due to the dominant contribution of the lithium counter electrode with FeF_3_ featuring relatively low volume change. The increase in the pressure change to areal capacity ratio from after the first cycle likely arises from progressive mechanical and electrochemical activation. During the first cycle, redistribution of reaction products or improved Li^+^ accessibility could increase the fraction of material participating in (de)­lithiation, leading to larger volume changes per unit capacity. With NMC counter electrodes (Figure 4f), the large normalized pressure change of the FeS_2_ in the first cycle is notable, which is reduced on later cycles.

### Microkinetic Modeling

To investigate the species evolution and reaction kinetics in solid-state conversion cathodes, we developed a mechanistic modeling framework to capture the electrode-scale reactions, transport processes, and electrochemical response of the cell. The electrochemical performance of the cell is strongly influenced by the physicochemical interactions and species evolution associated with the electrochemical reactions. Given the distinct active materials (sulfur, FeS_2_, and FeF_3_), the different species generated during each reaction lead to varying performance characteristics. The modeling framework couples reaction kinetics, charge transport, species evolution, and surface passivation arising from the formation of insulating Li_2_S and LiF, which restricts both ionic and electronic transport and impedes reaction reversibility. Butler–Volmer kinetics is used to capture the lithiation and delithiation behavior of the cells. The electrode microstructure is assumed to remain fixed during discharge, and the chemo-mechanical effects arising from volume changes associated with phase conversion are not included in the model. These coupled deformation driven effects remain an important direction for future work. The details of the model are explained in the Experimental section.

Based on previously reported studies,^33,57,60,65,66^ the reactions shown in Table 1 and their corresponding equilibrium potentials (vs Li/Li^+^) are assumed to occur in the cathode during lithiation and delithiation in the various cases considered in the model.

A comprehensive validation of the proposed model was conducted for all three cathode chemistries during both lithiation and delithiation processes at a current density of 0.1 mA cm^–2^, as illustrated in Figure 5a–c. These plots contain experimental data as well as simulated data from our model. The predicted voltage curves from the model demonstrate excellent agreement with the experimental curves, with similar voltage features corresponding to the various phase transitions, as well as similar reaction overpotentials.

**Figure 5:**
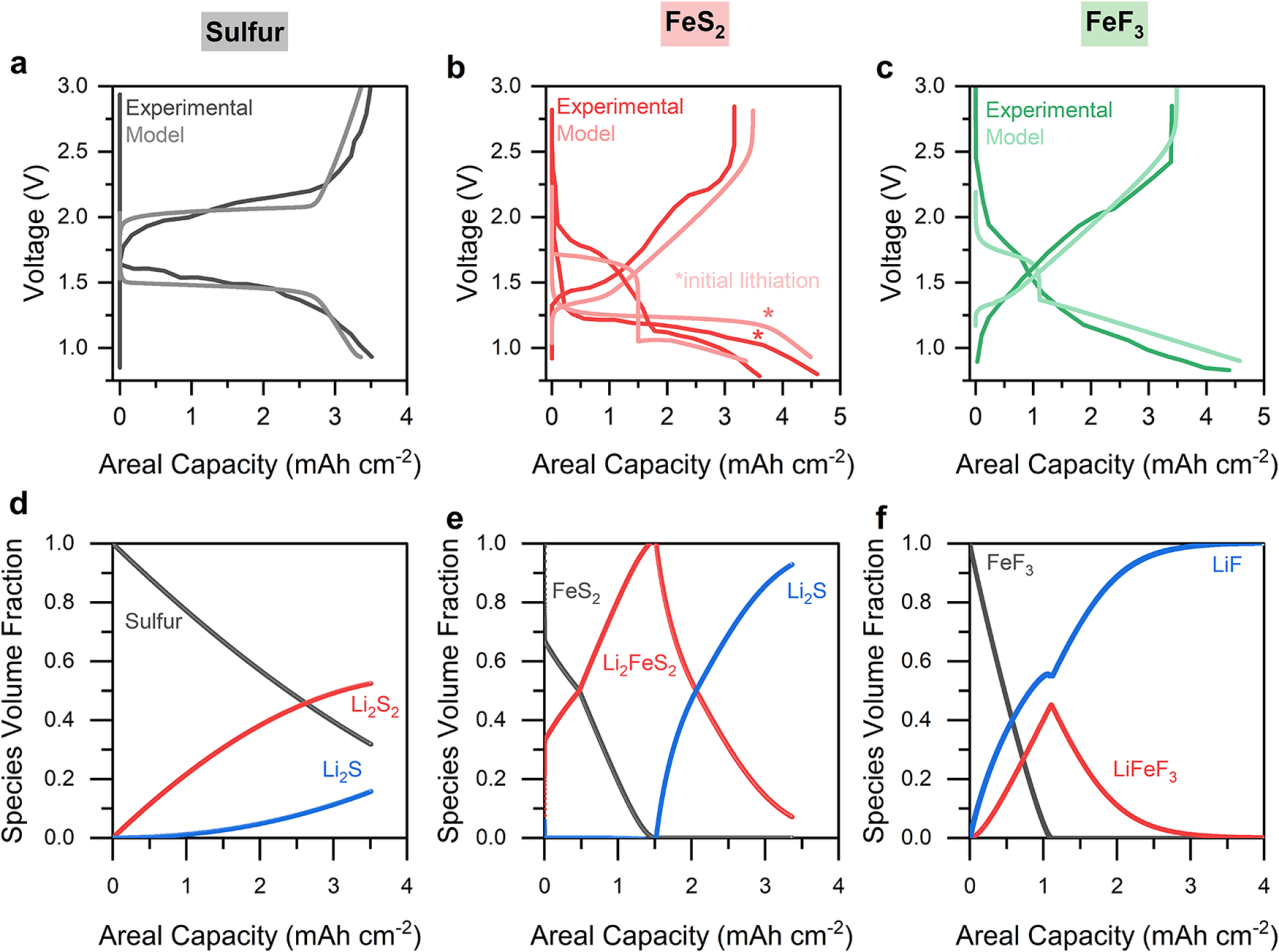
Comparison of the electrochemical behavior predicted by the model with experimental data for cells with (a) sulfur, (b) FeS_2_, and (c) FeF_3_ cathodes during both lithiation and delithiation processes. Two discharge curves (initial lithiation and first cycle) are highlighted for the FeS_2_ cathode demonstrating disparate reaction pathways after the first cycle. Consumption and generation of different phases during the lithiation process for (d) sulfur, (e) FeS_2_, and (f) FeF_3_ cathodes.

Reactions 9 and 11 (Table 1) depict the lithiation reaction pathway in a typical solid-state sulfur cathode, where Li_2_S_2_ forms as an intermediate during the conversion of elemental sulfur to Li_2_S.^57^ The close proximity of the equilibrium potentials for these reactions results in a spectrum of coexistence between Li_2_S_2_ and Li_2_S throughout the lithiation process. Figure 5d shows the phases present (as predicted by the model and based on the voltage curve) during lithiation. This plot illustrates that at the initial stage of lithiation, sulfur converts predominantly to Li_2_S_2_, with only minor Li_2_S formation. As lithiation progresses, Li_2_S_2_ further reacts to form Li_2_S (Figure S2). However, Li_2_S, being an electronic insulator, forms a passivating layer on the sulfur surface that obstructs electron percolation within the active material. As a result, 31.7% of sulfur remains unreacted, while only 15.8% is converted to Li_2_S and 52.5% to Li_2_S_2_. The buildup of Li_2_S leads to increased overpotentials and a characteristic voltage drop (Figure 1a), reflecting the kinetic hindrance imposed by the insulating lithiation (discharge) product.

However, the FeS_2_ and FeF_3_ cathodes exhibit distinct species evolution behavior during lithiation. In FeS_2_ cathodes, the significant difference between the equilibrium potentials of the two reactions results in FeS_2_ being fully converted to Li_2_FeS_2_ before Li_2_FeS_2_ is subsequently transformed into Li_2_S to complete the lithiation reaction (Figure 5e). In the FeF_3_ cathode, FeF_3_ is initially converted to LiFeF_3_, and due to the relatively small equilibrium potential difference with the second reaction, LiFeF_3_ simultaneously converts to LiF during the lithiation process (Figure 5f). Although Li_2_S also forms during FeS_2_ conversion, and LiF in the case of FeF_3_ exhibits similar insulating behavior, the concurrent *in situ* formation of metallic Fe in both of these cases provides electronic pathways that sustain reaction front propagation. This mechanism enables more complete conversion of FeS_2_ and FeF_3_, resulting in a higher percentage of active material utilization compared to the sulfur cathode (Figure 5e,f).

The distinct electrochemical characteristics of the active materials and their lithiation products dictate the extent of active material consumption and the spatial evolution of species within the electrode (Figure 6). To ensure consistent areal capacity, the electrode thicknesses were set to 75 μm for sulfur, 90 μm for FeS_2_, and 120 μm for FeF_3_. Across all three cathode chemistries, the lithiation process induces moderate concentration gradients, primarily due to the limited transport of Li^+^ ions through solid–solid contact interfaces within the composite electrode. Unlike liquid electrolytes, where ion transport is rapid and spatially uniform, solid-state systems rely on point or surface contacts between particles for ion conduction. This restricted transport confines lithiation predominantly to regions adjacent to the separator–cathode interface, where lithium availability is highest. As lithiation of the active material progresses, the reaction front gradually advances inward as ionic accessibility permits, leading to a spatially resolved reaction zone within the cathode.^67,68^

**Figure 6:**
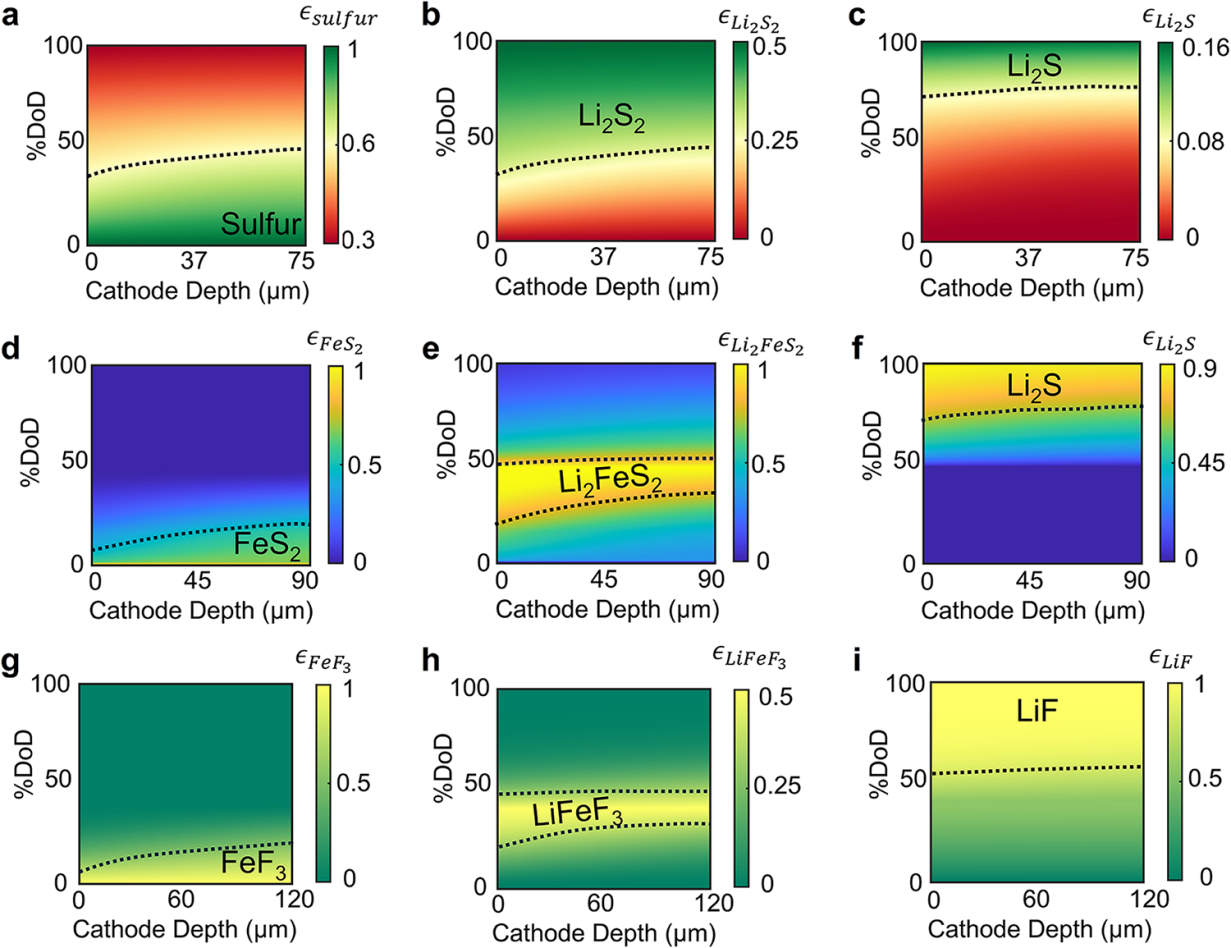
Spatiotemporal evolution of species in (a–c) sulfur, (d–f) FeS_2_, and (g–i) FeF_3_ composite cathodes as a function of depth of discharge (DoD). Here, the vertical axis tracks lithiation progression, the horizontal axis denotes cathode depth, and ϵ*_i_* denotes the volume fraction of species *i*.

Interestingly, the initial concentration gradients diminish across all three cathode systems toward the end of the lithiation (Figure 6). This homogenization arises from the progressive deactivation of reaction sites near the separator interface by insulating lithiation products, such as Li_2_S or LiF, which impede further electrochemical reactions at these locations. As a result, the current density redistributes toward deeper regions of the electrode that remain electrochemically active. This redistribution promotes the advancement of the lithiation front until the remaining unreacted material becomes limited by ionic transport or is passivated by insulating phases. Consequently, the lithiation process transitions from being reaction-kinetics-controlled at the interface to being transport-limited within the electrode bulk.

The differing molar volumes and fractions of active materials and lithiation products give rise to distinct degrees of cathode expansion, governed by both reaction stoichiometry and the physical properties of the phases involved. We estimate the extent of expansion by combining the molar volume and species fraction of each phase, assuming constant microstructural properties and cathode thickness during volume change (See Experimental section). In the sulfur cathode,^19^ the molar volumes of Li_2_S_2_ and Li_2_S are approximately 60 and 80% greater than that of sulfur, respectively, leading to a 26% increase in cathode volume by the end of lithiation due to mixed product formation (Figure S3). FeS_2_ exhibits the highest expansion (110%), followed by sulfur (26%) and FeF_3_ (21%), reflecting variations in both product volumes and conversion extents.

These results suggest that volume expansion is not merely a mechanical effect but a critical factor that could influence interfacial contact, reaction front propagation, and electrochemical performance. Therefore, understanding and managing cathode expansion remains essential for the design of stable and high-performance solid-state conversion cathodes. Since solid–solid contact area and cathode thickness inevitably evolve with expansion and contraction, accounting for microstructural changes is a critical direction for future work.

We next investigated the morphological evolution of cathode composites using cross-sectional scanning electron microscopy (SEM) to assess crack formation, void development, and delamination (Figures S4–5). Initially, all pristine pellets exhibited compact and dense microstructures (Figure S4). However, after one electrochemical cycle, degradation was observed across all samples, including cracking and delamination. This degradation is primarily attributed to the large volume changes associated with conversion-type reactions during lithiation and delithiation. The severity of these structural changes underscores the mechanical instability of these cathode materials under realistic operating conditions. Moreover, the complex reaction pathways of these materials, often involving multiple intermediate phases, may contribute to nonuniform expansion, further exacerbating the mechanical stress. The magnified SEM images of the pristine and delithiated state of the various cathode composites are shown in Figure S5, showing that all undergo morphology changes after one cycle.

### X-ray Absorption Spectroscopy

In comparison to sulfur, the reaction mechanisms of FeS_2_ and FeF_3_ are complicated by the chemo-mechanical interactions of iron species with the lithium–sulfur species, as well as the formation of a variety of reaction intermediates and products during cycling.^13,69,70^ To analyze the formation of iron species with different oxidation states, we performed *ex situ* XAFS measurements at the Fe K-edge for FeS_2_ and FeF_3_ electrode composites using pristine, lithiated and delithiated pellets (Figure 7). The X-ray absorption near edge structure (XANES) region captures the valency and electronic configuration of the iron species. The Fe K-edge positions were determined using the half-height method (Table S1), where the edge is defined as the energy at which the normalized absorption reaches 50% of the maximum intensity (Figure 7a,b, insets). For FeS_2_, the edge shifts slightly to lower energy in the lithiated state, indicating the formation of Li_2_FeS_2_ with Fe^2+^ as well as metallic iron particles.^71^ It is known that FeS_2_ tends to form nanoscale metallic iron particles at the end of discharge, which may explain the absence of distinct Fe shells in the R-space spectrum.^72^ Upon delithiation, the edge shifts slightly to higher energy, suggesting the formation of species different from the initial FeS_2_, consistent with electrokinetic modeling and confirming the formation of FeS. While both FeS_2_ and FeS contain Fe^2+^, their electronic structures differ substantially.^73^ The FeF_3_ material exhibits different behavior. The lower Fe K-edge of FeF_3_ is attributed to the reaction between FeF_3_ and Li_6_PS_5_Cl that promotes formation of FeS that shifts the K-edge to the lower energy range.^15,74^ The Fe K-edge shifts by approximately 0.5 eV to lower energy upon discharge, confirming lithiation. After charging, the edge returns to its original position, indicating reversible delithiation.

**Figure 7:**
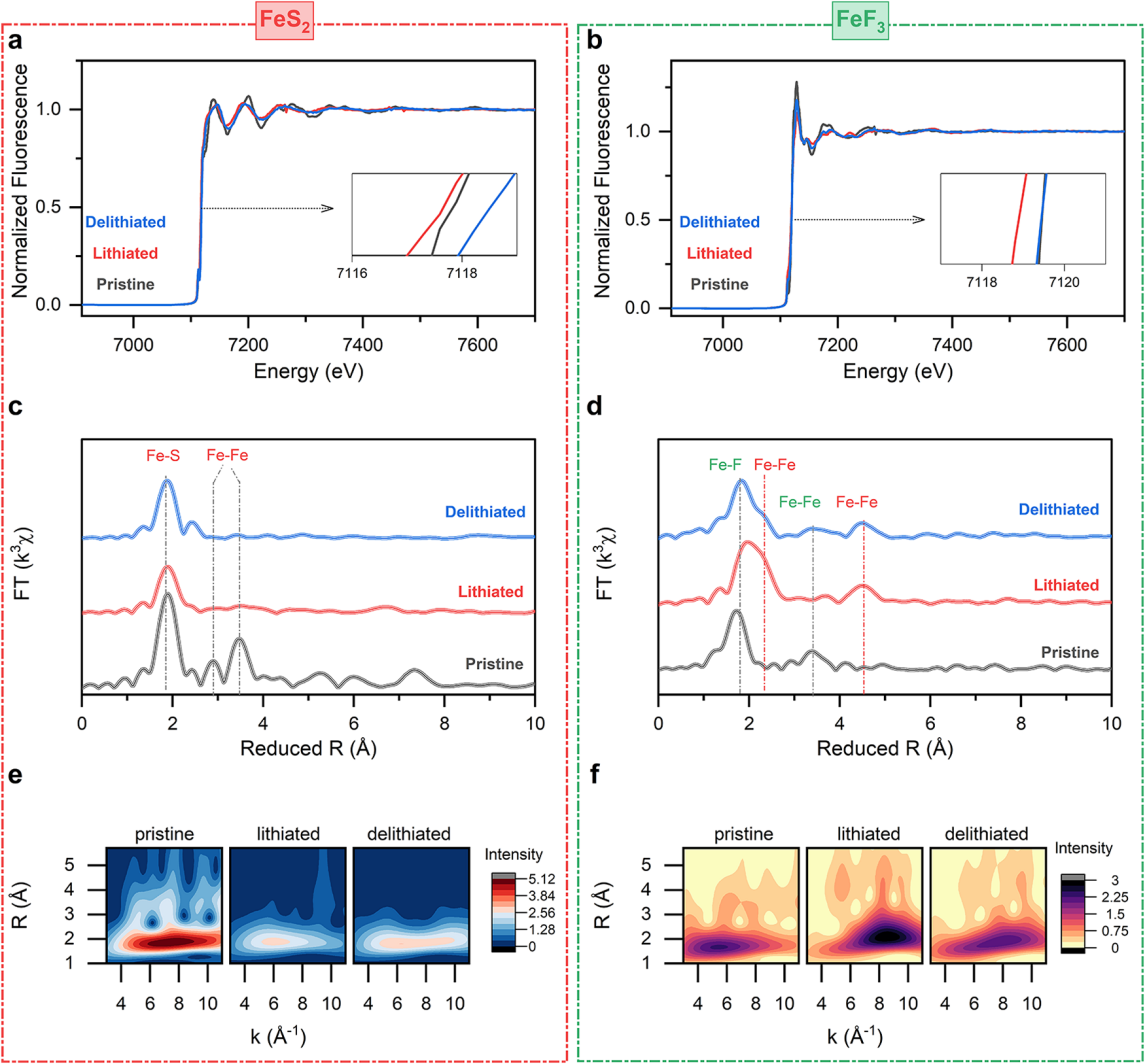
Ex situ X-ray absorption spectroscopy analysis of FeS_2_ and FeF_3_ composites. Fe K-edge X-ray absorption near-edge (XANES) spectra of (a) FeS_2_ and (b) FeF_3_. Extended X-ray absorption fine-structure of (c) FeS_2_ and (d) FeF_3_ composite cathodes. WT-EXAFS of (e) FeS_2_ and (f) FeF_3_ composite cathodes in the pristine state (black), after the initial lithiation (red) and after the first delithiation (blue).

Fourier-transformed (Figures 7c,d and S6–7) and wavelet-transformed (Figure 7e,f) extended X-ray absorption fine structure (EXAFS) spectra for FeS_2_ and FeF_3_ reveal distinct coordination environments across different states of charge. Fitting of the Fourier-transformed spectra confirms the presence of Li_2_FeS_2_ even at the end of the first lithiation reaction and the formation of FeS after delithiation. In FeF_3_, changes in Fe–F coordination are observed around 2 Å, along with the appearance of new coordination shells near 4.3 Å, indicative of metallic iron formation.^75^ The irreversible chemical evolution of FeS_2_ correlates with the asymmetric pressure hysteresis observed during cycling. Such irreversible phase transitions generate internal stress and contribute to chemo-mechanical degradation. In contrast, these measurements show that FeF_3_ exhibits more reversible redox processes, and it is reflected in the relatively symmetric cyclic pressure profiles that suggest less structural disruption and lower residual stress accumulation. Wavelet-transformed EXAFS (WT-EXAFS) provides a two-dimensional representation of the EXAFS signal in both *R* and *k* space, allowing the separation of overlapping scattering paths and enabling the identification of different neighboring atoms that are otherwise indistinguishable in conventional Fourier-transformed spectra. The WT spectra of both FeS_2_ (Figure 7e) and FeF_3_ (Figure 7f) display clear variations in energy space, reflecting changes in the local coordination environment of iron sites during cycling. We note that the *ex situ* XAS measurements presented here capture only the final reaction states after full lithiation or delithiation and therefore cannot directly resolve transient intermediate phases that may form during cycling. Nevertheless, the presence and evolution of such intermediates can be inferred through a combination of electrochemical signatures, stack pressure evolution, and observed mechanistic pathways reported in prior studies.^60,65,66^ Specifically, the nonlinear and stepwise pressure changes observed during the early stages of lithiation are consistent with sequential phase evolution. Future *in situ* XAS or *operando* diffraction studies would further resolve the real-time evolution of these phases and provide direct validation of the inferred mechanisms.

## Conclusions

This study investigates the electro-chemo-mechanical behavior of sulfur, FeS_2_, and FeF_3_ conversion cathode composites in solid-state batteries. These materials undergo varying degrees of volume expansion and contraction during cycling. We demonstrate that volume changes in the counter electrode can significantly influence the overall volume evolution of the cell stack. All three cathodes exhibit pressure profiles with changing slopes during lithiation and delithiation, varying across cycles. Our findings link pressure changes to the formation of reaction intermediate and final products via microkinetic modeling, correlating these with microstructural evolution. Spectroscopic analyses reveal that FeS_2_ and FeF_3_ possess distinct iron coordination environments, resulting in characteristic differences in their microstructural transformations.

This work establishes a connection between microstructural evolution and the chemo-mechanical behavior of these materials. Looking forward, further investigation of the reaction mechanisms, along with *operando* imaging and spectroscopy, is essential to fully understand and address the chemo-mechanical challenges in these systems. Investigation at reduced stack pressures is also necessary, as this is relevant to practical devices. Low stack pressures may exacerbate loss of interparticle contact and accelerate capacity fade, particularly if electrode porosity is not carefully optimized. Porous architecture of the composite cathodes can help accommodate volume fluctuations but must balance mechanical properties and ionic and/or electronic transport pathways. Understanding the interplay between volume change, porosity, and stack pressure is key for designing stable, high-capacity conversion-type cathodes for solid-state batteries.

## Supplementary Material



## Data Availability

All data of this study are available from the corresponding author upon reasonable request.

## Notes

XANES and EXAFS modeling results. Supporting figures include **Supporting Figure S1**. Electrochemical behavior of sulfur, FeS_2_, and FeF_3_ cathodes cycled with NMC counter electrodes. **Figure S2**. Microstructural evolution during discharge. The left panel shows the initial stage where sulfur begins to convert to Li_2_S_2_. The middle panel corresponds to an intermediate state where sulfur is partially converted to Li_2_S_2_ and Li_2_S_2_ begins transitioning to Li_2_S. The right panel represents the discharged state containing a mixed Li_2_S_2_ and Li_2_S product distribution. **Figure S3**. Volume expansion for sulfur, FeS_2_ and FeF_3_ cathodes during the lithiation process. **Figures S4–5**. SEM images of pristine electrodes (a-c) and electrodes after one cycle (in the delithiated state) (d-f) of sulfur, FeS_2_, and FeF_3_ cathodes, respectively. **Figure S6**. k-space X-ray absorption fine-structure spectra of FeS_2_in the pristine, discharged, and charged states at the Fe K-edge. **Figure S7**. k-space X-ray absorption fine-structure spectra of FeF_3_ in the pristine, discharged, and charged states at the Fe K-edge. (PDF)
