# Supplementary material for: Linking Pressure to Electrochemical Evolution in Solid-State Conversion Cathode Composites
Source: ACS Appl Mater Interfaces. 2025 Dec 31;18(1):1626–40. doi: 10.1021/acsami.5c20956 (PMC12781064; doi:10.1021/acsami.5c20956)
Supplement: Supplementary file 1 [file am5c20956_si_001.pdf]

Supporting Information

Linking Pressure to Electrochemical Evolution in Solid-State Conversion Cathode Composites

Elif Pınar Alsaç[1], Arpan Kumar Sharma[2], Sun Geun Yoon[1], Bairav S. Vishnugopi[2], Congcheng Wang[1], Talia A. Thomas[1], Douglas Lars Nelson[3], Udochukwu D. Eze[3], Won Joon Jeong[3], John Harris[3], Partha P. Mukherjee[2], Matthew T. McDowell[1,3]*

[1] George W. Woodruff School of Mechanical Engineering, Georgia Institute of Technology, Atlanta, GA, USA.

[2] School of Mechanical Engineering, Purdue University, West Lafayette, IN, USA.

[3] School of Materials Science and Engineering, Georgia Institute of Technology, Atlanta, GA, USA.

Corresponding Author: mattmcdowell@gatech.edu

Table S1. Location of Fe K-edges from XANES spectra on FeS_2 and FeF_3

	**FeS_2**	**FeF_3**
Pristine	7117.77 eV	7119.44 eV
Discharged	7117.62 eV	7118.86 eV
Charged	7118.45 eV	7119.41 eV

Table S2. EXAFS simulation parameters of Fe K-edge of FeS_2 composites

Sample	s^2_S	s^2_{Fe}	E_0	R_{Fe-S}	N	R_{Fe-Fe}	N	R-Factor
Pristine	0.003	0.0047	8.559+/- 1.05	2.262<br>3.443<br>3.610	6<br>6<br>2	3.827	12	0.038
Discharged	0.005	0.008	7.677+/-1.15	2.030	3.7	2.6808	1.2	0.028
Charged	0.004	0.009	9.102 +/- 0.8877	2.264	3.8	2.731	1.2	0.014
S_0^2=0.7, $E_{0,Fe}$= 7112 eV								

Table S3. EXAFS simulation parameters of Fe K-edge of FeF_3 composites

Sample	s^2_F	s^2_{Fe}	E_0	R_{Fe-F}	N	R_{Fe-Fe}	N	R-Factor
Pristine	0.006	0.011	12.273+/-0.883	2.073	6	3.791	6	0.023
Discharged	0.005<br>0.005	0.015<br>0.007<br>0.007	12.175+/-0.657	2.200<br>2.180	3<br>1.5	3.652<br>2.367<br>3.106	4.5<br>9<br>4	0.045
Charged	0.005	0.014<br>0.011<br>0.015	14.487+/-1.675	2.111	5	3.748<br>2.385<br>3.676	5<br>6<br>3	0.037
S_0^2=0.5, $E_{0,Fe}$= 7112 eV								

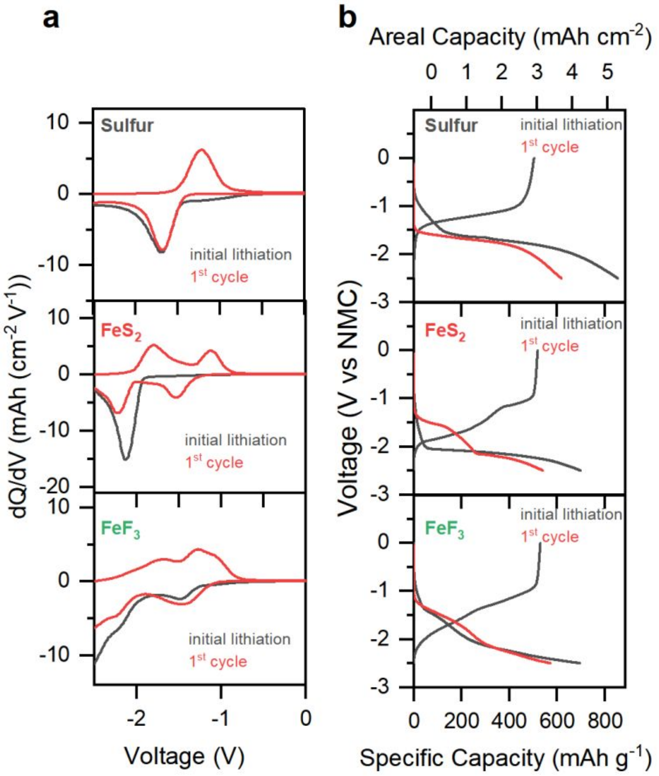

Figure S1. Electrochemical behavior of sulfur, FeS_2, and FeF_3 cathodes cycled with NMC counter electrodes. The experiments were carried out at room temperature. **a)** Differential capacity (dQ/dV) curves of sulfur, FeS_2, and FeF_3 cathode composites. All cells were cycled at room temperature. **b)** The corresponding charge-discharge profile for each cell in panels a-c.

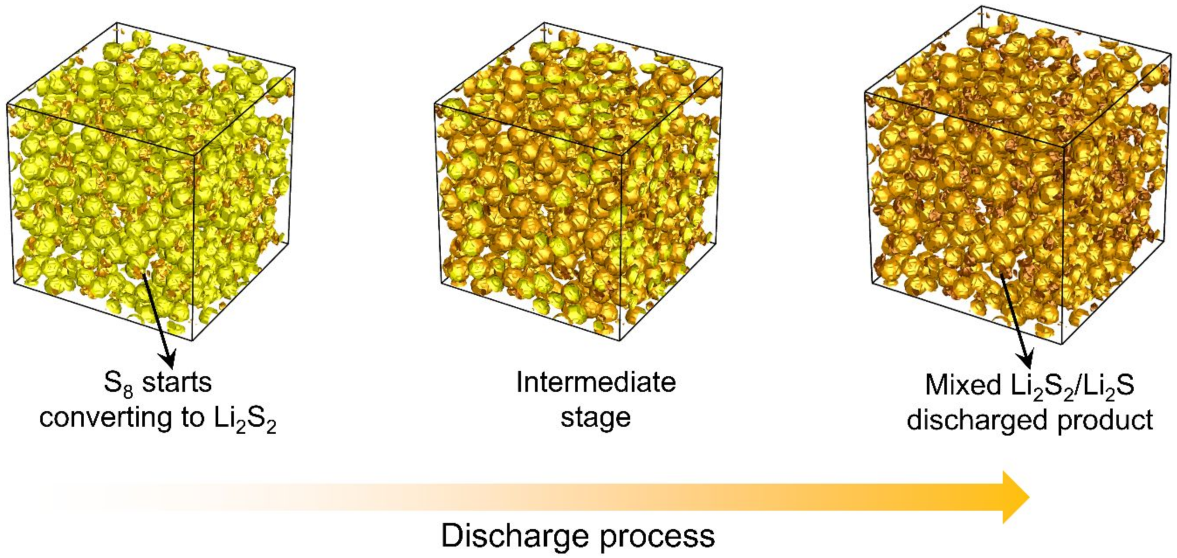

Figure S2. Microstructural evolution during discharge. The left panel shows the initial stage where sulfur begins to convert to Li_2S_2. The middle panel corresponds to an intermediate state where sulfur is partially converted to Li_2S_2, and Li_2S_2 begins transitioning to Li_2S. The right panel represents the discharged state containing a mixed Li_2S_2 and Li_2S product distribution.

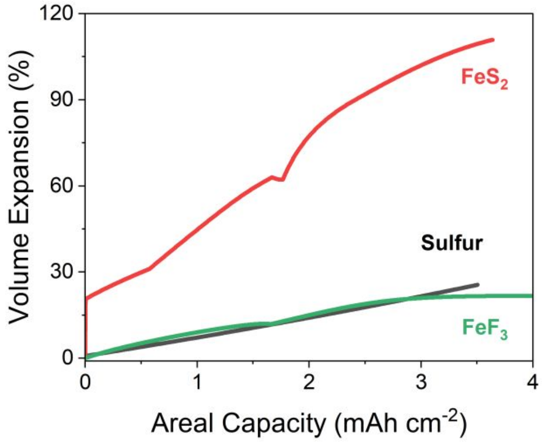

Figure S3. Volume expansion for sulfur, FeS_2, and FeF_3 composite cathodes during the lithiation process.

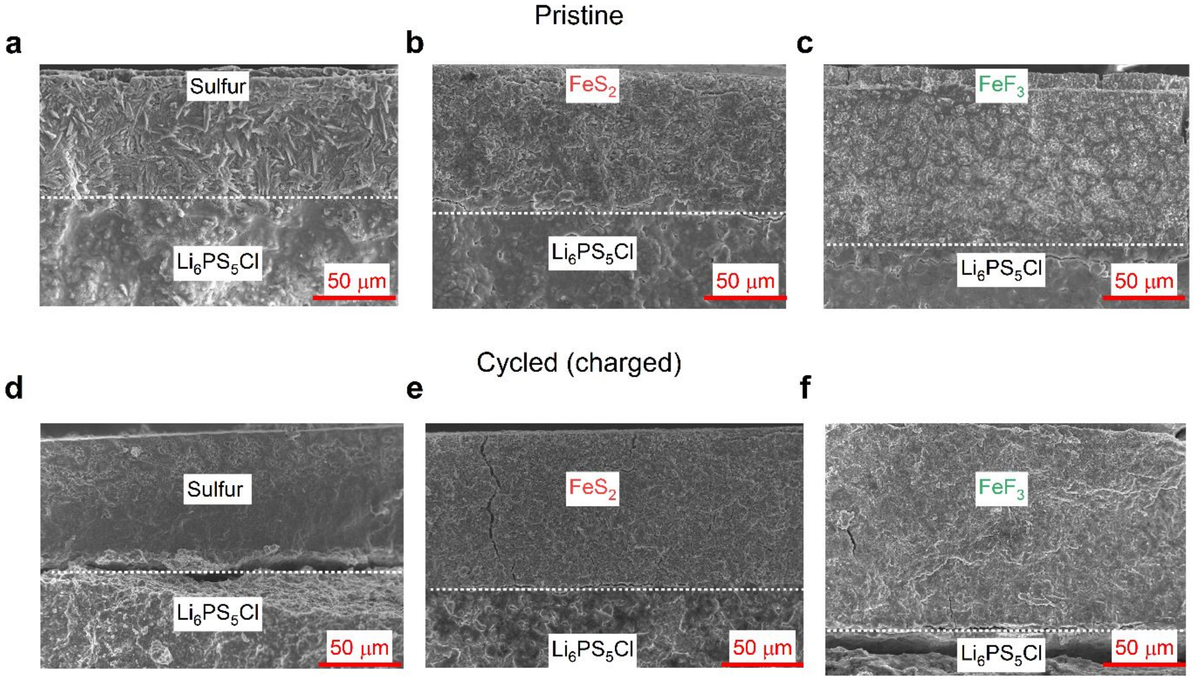

Figure S4. Cross-sectional SEM images of sulfur, FeS_2, and FeF_3 composite electrodes in the pristine state (a-c) and after one cycle in the charged state (d-f). To maintain consistent areal capacity across the different materials for the stack pressure experiments, the electrode thicknesses were adjusted according to the theoretical specific capacities of the active materials. Therefore, the sulfur composite had the lowest (75 mm) and FeF_3 had the highest (120 mm) thickness.

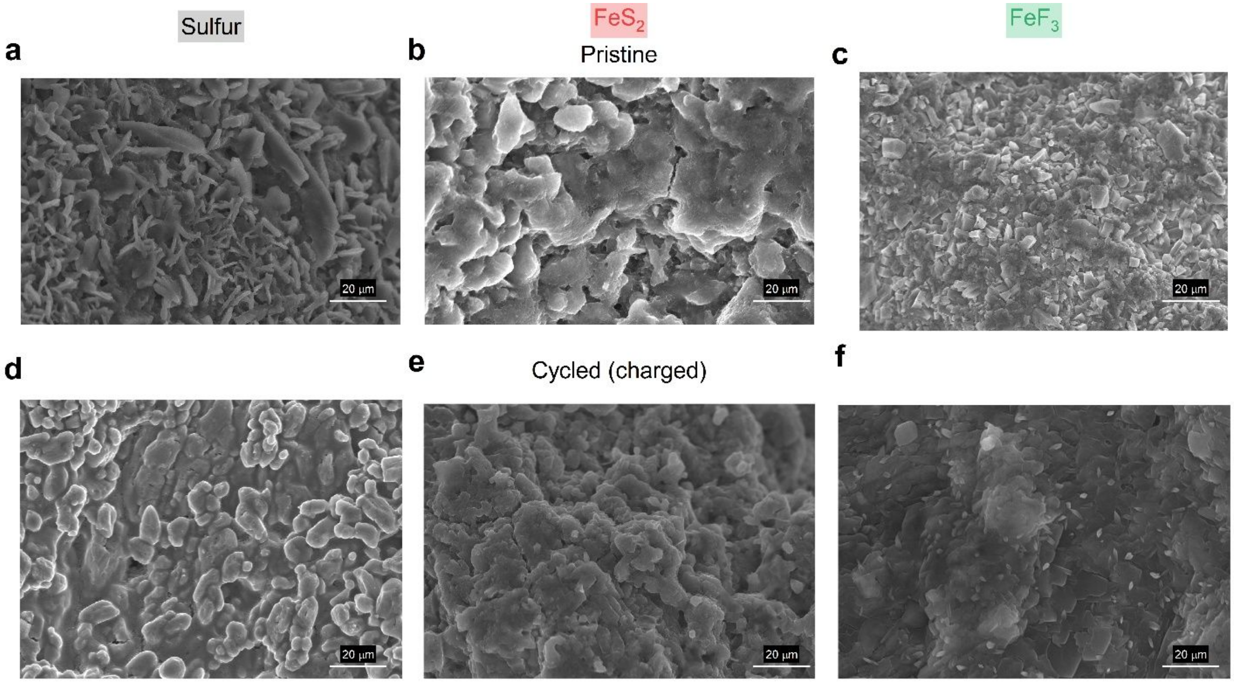

Figure S5. Magnified SEM images of pristine electrodes (a-c) and electrodes after one cycle (in the delithiated state) (d-f) of sulfur, FeS_2, and FeF_3 cathodes, respectively.

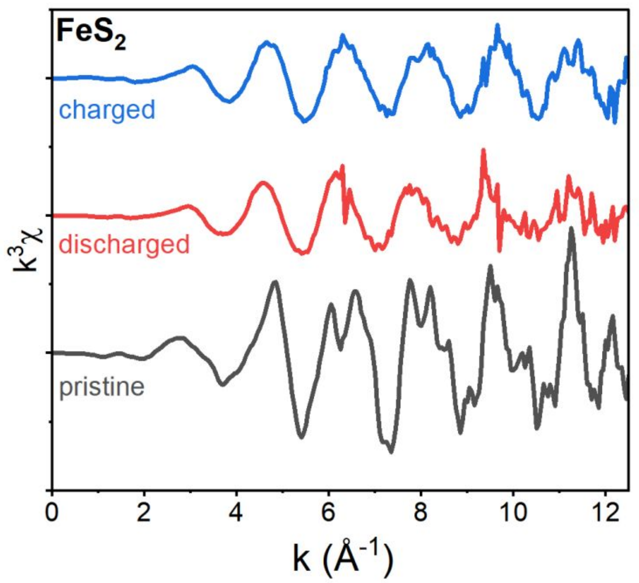

Figure S6. k-space X-ray absorption fine-structure spectra of FeS_2 in the pristine, discharged, and charged states at the Fe K-edge.

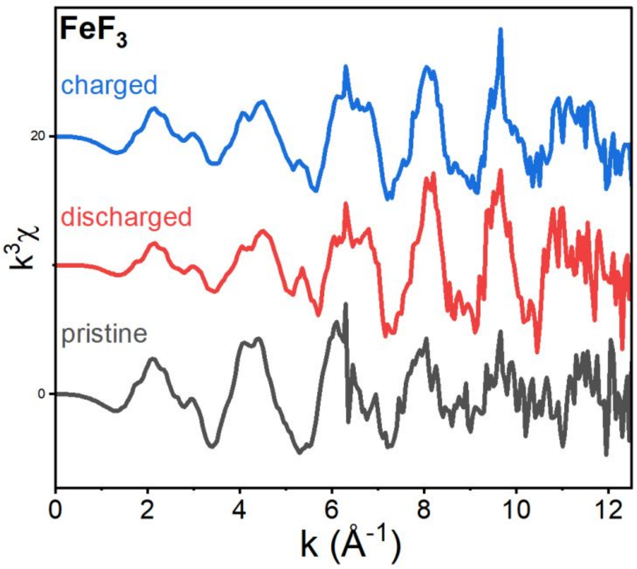

Figure S7. k-space X-ray absorption fine-structure spectra of FeF_3 in the pristine, discharged, and charged states at the Fe K-edge.
